# Turn-On Fluorescence Probe for Cancer-Related *γ*-Glutamyltranspeptidase Detection

**DOI:** 10.3390/molecules29194776

**Published:** 2024-10-09

**Authors:** Muhammad Saleem, Muhammad Hanif, Samuel Bonne, Muhammad Zeeshan, Salahuddin Khan, Muhammad Rafiq, Tehreem Tahir, Changrui Lu, Rujie Cai

**Affiliations:** 1 Shanghai Key Laboratory of Plant Molecular Sciences, College of Life Sciences, Shanghai Normal University, Shanghai 200234, China; 2 Department of Chemistry, Thal University Bhakkar, Bhakkar 30000, Pakistan; 3 Department of Chemistry, University of Sargodha, Sargodha 40100, Pakistan; 4 Department of Chemistry, GC University Faisalabad, Sub Campus, Layyah 31200, Pakistan; 5 Faculty of Medicine, McGill University, Montreal, QC H3A 0G4, Canada; samuel.bonne@mail.mcgill.ca; 6 College of Engineering, King Saud University, P.O. Box 800, Riyadh 11421, Saudi Arabia; drskhan@ksu.edu.sa; 7 Department of Physiology and Biochemistry, Cholistan University of Veterinary and Animal Sciences, Bahawalpur 6300, Pakistan; 8 Department of Biosciences, College of Biological Sciences and Medical Engineering, Donghua University, 2999 North Ren Min Road, Shanghai 201620, China; crlu@dhu.edu.cn

**Keywords:** fluorescence, fensor, *γ*-glutamyltranspeptidase, molecular docking, bioimaging

## Abstract

The design and development of fluorescent materials for detecting cancer-related enzymes are crucial for cancer diagnosis and treatment. Herein, we present a substituted rhodamine derivative for the chromogenic and fluorogenic detection of the cancer-relevant enzyme *γ*-glutamyltranspeptidase (GGT). Initially, the probe is non-chromic and non-emissive due to its spirolactam form, which hinders extensive electronic delocalization over broader pathway. However, selective enzymatic cleavage of the side-coupled group triggers spirolactam ring opening, resulting in electronic flow across the rhodamine skeleton, and reduces the band gap for low-energy electronic transitions. This transformation turns the reaction mixture from colorless to intense pink, with prominent UV and fluorescence bands. The sensor’s selectivity was tested against various human enzymes, including urease, alkaline phosphatase, acetylcholinesterase, tyrosinase, and cyclooxygenase, and showed no response. Absorption and fluorescence titration analyses of the probe upon incremental addition of GGT into the probe solution revealed a consistent increase in both absorption and emission spectra, along with intensified pink coloration. The cellular toxicity of the receptor was evaluated using the MTT assay, and bioimaging analysis was performed on BHK-21 cells, which produced bright red fluorescence, demonstrating the probe’s excellent cell penetration and digestion capabilities for intracellular analytical detection. Molecular docking results supported the fact that probe-4 made stable interactions with the GGT active site residues.

## 1. Introduction

Despite extensive efforts in cancerous imaging and therapy, cancer continues to be a major global health concern. Early and accurate detection, along with effective treatment, are essential for reducing fatality and improving patient outcomes [1,2,3]. The high mortality rate due to cancer is primarily due to late diagnosis and limited treatment options [4,5,6,7,8,9]. Small-molecule fluorescent sensors exhibited a prominent role in the sensation and imaging of cancer-related enzymes. Fluorescence imaging technology is highly valued for its specificity, sensitivity, non-invasiveness, and cost-effectiveness [10,11].

There are several tumor-related enzymes that are often used as biomarkers for cancer diagnosis, including alkaline phosphatase [12], aminopeptidase, cyclooxygenase [13], glycosidase [14], proteases [15] and *γ*-glutamyltranspeptidase [16]. Enzymes are essential natural biomarkers, facilitating over 5000 specific biochemical reactions. *γ*-glutamyltranspeptidase is the enzyme located on the cellular surface and is a critical biomarker due to its role in *γ*-glutamate cleavage in glutathione and related compounds. It is overexpressed in various cancers, including liver, ovarian, and cervical tumors [17]. GGT maintains cellular redox equilibrium and promotes tumor proliferation and drug resistance. Additionally, it is associated with atherosclerosis and is a conventional indicator in liver disease diagnosis in LFT tests. Elevated GGT levels are linked to oxidative stress, cancer cell metabolism, and drug resistance. High GGT levels in plasma can indicate viral hepatitis, bone disease, and inflammatory bowel disease [18,19,20].

Researchers have focused on the fluorescence probe using rhodamine B as fluorophore due to its red region low-energy intense optical properties. In contrast to conventional analytical detection techniques like atomic absorption spectroscopy, atomic fluorescence spectrometry, dispersive liquid–liquid microextraction, inductively coupled plasma atomic emission spectrometry, electrochemical sensing, and piezoelectric quartz crystal use, fluorescence spectroscopic methods offer notable advantages. These include non-invasiveness and energy-efficient sensitivity for monitoring the electronic environment before and after interactions with target analytes, making them valuable in fields such as biological imaging, drug screening, and biomarker-based medical diagnostics [21,22,23,24]. In the past year, several fluorescence probes with the fascinating enzymatic-triggered fluorescence response have been reported based on 1,8-naphthalimide [25], hemicyanines-resorufin [26], yridineacetonitrile [27] and benzothiazole [28]. Herein we have reported the synthesis of rhodamine B derivative 4, which acts as a fluorescence-reporting material for GGT in the solution as well as inside the cellular media. The probe–enzyme interaction was further investigated by the molecular docking analysis. The reported cells’ viable fluorescence probe with appreciable emissive and chromogenic properties might be useful for the detection and identification of cancer and related health risks.

## 2. Results and Discussions

The characterization of the probe was carried out by physical identification, FT-IR, and NMR spectroscopic analysis. There were prominent vibrational bands at 3675, 3445, and 3372 cm^−1^ due to the stretching vibrations of the primary amine and hydroxyl groups present in the probe. The higher-wavenumber peaks in the range of 2800 to 3181 cm^−1^ were due to the aromatic carbon-to-hydrogen bond vibrations. The carbonyl group appears as a high-intensity broadened peak at 1695 cm^−1^, and this signal was assigned to the urea carbonyl of the probe. The carbon-to-nitrogen double bond appears at 1998 and 1581, while the carbon-to-carbon double bond vibrates at 1548, 1490, 1455, and 1415 cm^−1^. The methoxy carbon-to-oxygen bond of the anisole moiety stretching vibration appeared as an intense sharp signal at 1236 cm^−1^. The proton NMR spectrum of the probe exhibited six signals in the aromatic regions, and all these peaks belong to the protons of the rhodamine moiety. The alpha hydrogen of the signaling unit appears as multiplets at 3.25 ppm. The two signals, one at 3.25 ppm as a triplet and other at 3.08 ppm as a multiplet, each with the multiplicity of two protons, were assigned to the signaling unit –CH_2_ groups. The highest intensity triplet with the multiplicity of 12 protons was due to the rhodamine terminal cage unit CH_3_.

### 2.1. Optical Analysis

**Figure 1:**
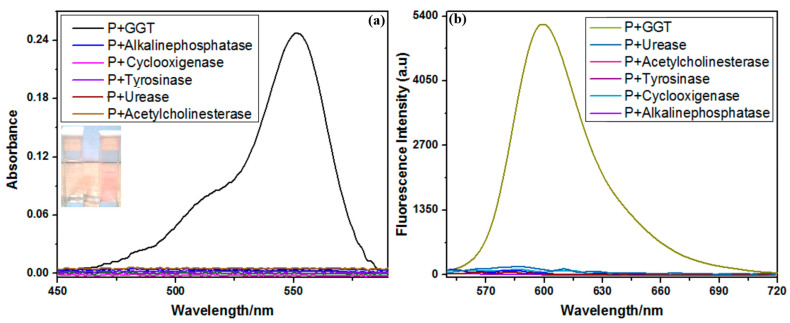
(**a**) UV and (**b**) fluorescence spectra of probe alone and after reaction with *γ*-glutamyltranspeptidase, urease, alkaline phosphatase, acetylcholinesterase, tyrosinase, and cyclooxygenase; inset represents chromogenic change in probe solution upon reaction with enzyme.

**Scheme 1:**
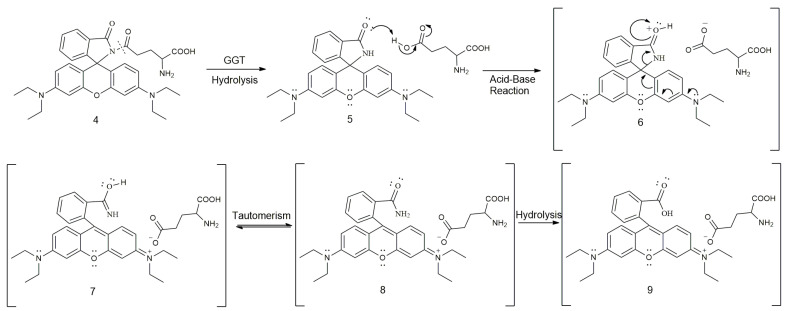
Sensing mechanism of probe toward GGT.

The absorption and fluorescence spectral measurements of the probe before and after treatment with *γ*-glutamyltranspeptidase, urease, alkaline phosphatase, acetylcholinesterase, tyrosinase, and cyclooxygenase were obtained in aqueous:methanol, 60:40, *v*/*v* %, as shown in Figure 1. The probe, both alone as well as upon treatment with urease, alkaline phosphatase, acetylcholinesterase, tyrosinase, and cyclooxygenase, was non-emissive and colorless, with no UV and fluorescence at 550–750 and 450–600 nm, respectively. The non-emissiveness of the probe was due to its existence in the spirolactam form, which hinders the electronic delocalization over the longer pathway [29,30] (Scheme 1).

However, an abrupt increment in absorption and emission was observed along with a chromogenic transformation in the reaction mixture to a pinkish shade when the reaction mixture was operated for optical analysis before incubation with GGT for 30 min at 37 degrees Celsius. These responses of the probe were utilized as a GGT-sensing strategy in the mixed aqueous–organic media.

**Figure 2:**
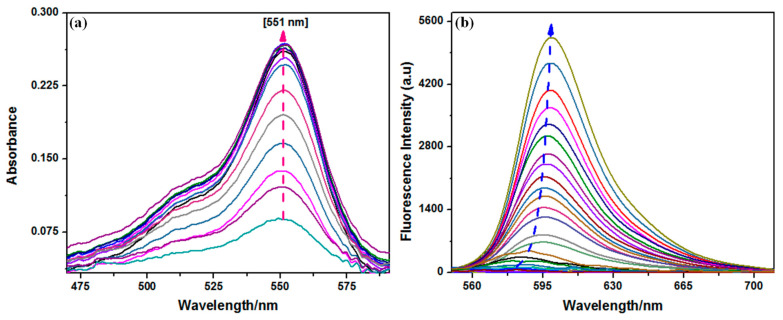
(**a**) UV and (**b**) fluorescence titration of probe upon incremental induction of GGT (10–100 µL from 0.05 U/mL enzyme solutions) into probe solution (30 µM).

The absorption and fluorescence titration analysis of the probe was performed upon the incremental induction of the GGT (10–100 µL from 0.05 U/mL enzyme solutions) into the probe solution (30 µM). Both the absorption and the emission response of the probe were directly proportional to the enzyme concentration and became saturated at 100 µL enzymatic inductions (Figure 2). The enhancement of the UV-visible signal was smooth, with increment in the intensity of the signal at absorption maxima of 551 nm. However, the fluorescence spectra of the probe exhibited a sudden red shift upon initial enzymatic reaction, while afterwards it became smooth with the increment of emission intensity at emission maxima of 598 nm. These titration analyses also caused pink color intensification in the reaction mixture, which might be employed as a bare-eye enzymatic detection from the solution.

**Figure 3:**
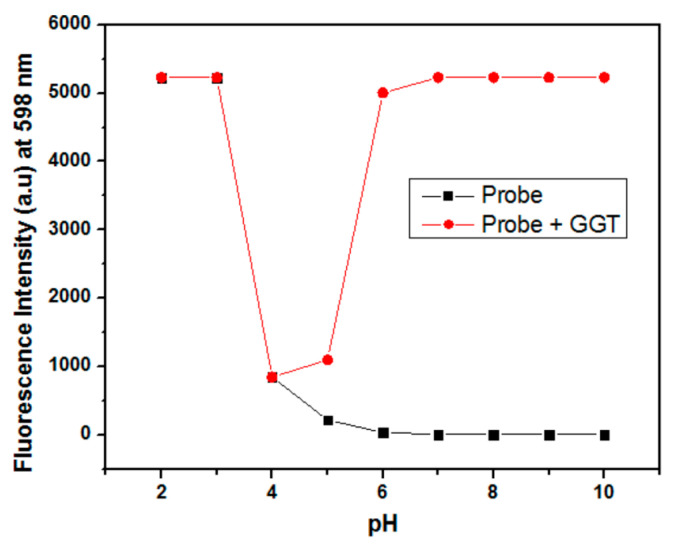
pH tolerance of probe and probe–GGT solution.

The probe was observed to be functional toward GGT detection in the broad pH range of pH 4–10. The probe–GGT solution exhibited a maximal emission response in the pH ranging from 6 to 10; however, the emission signal was also harvested with appreciable intensity at pH 4 and 5 as well. In the strong acidic media, the probe was unstable and underwent acid-induced intramolecular ring opening of the spirolactam skeleton, which turned the electronic delocalization and flow over the longer area, enabling the operation of a low-energy electronic transition in the visible regions of the electromagnetic spectra (Figure 3).

### 2.2. Solvent Effect

**Figure 4:**
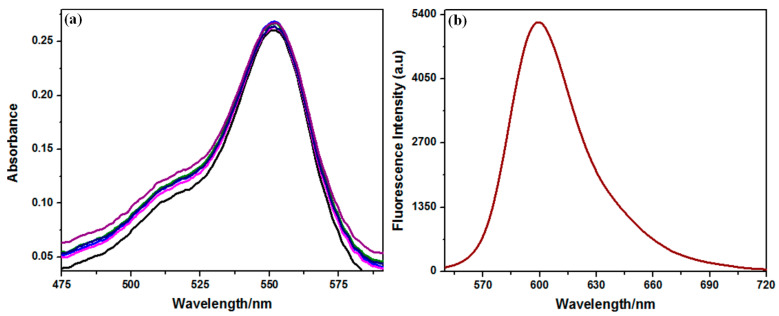
(**a**) UV and (**b**) fluorescence analysis of probe–GGT mixture in varieties of pure organic and mixed aqueous–organic solvent systems.

Solvent tolerance by the probe was assessed by using varieties of pure organic and mixed solvents. The probe exhibited a very good response in DMSO, ethanol, and methanol, while a similar response was recorded in the case of the mixed aqueous and organic solvent system. However, the probe molecules precipitated in the pure aqueous solvents. The slight variation in the peak height of the probe–analyte mixture was found by using varieties of solvents without any spectral shifting (Table 1). Meanwhile, these spectroscopic data have been represented graphically as well in Figure 4.

### 2.3. Molecular Docking

**Figure 5:**
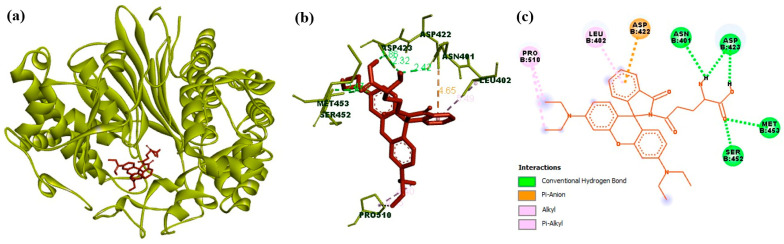
(**a**) Docked complex of GGT (PDB: 4ZBK) and probe-4 with minimum binding energy (kcal/mol); (**b**) 3D representation of key interacting groups between GGT and probe-4 with distances in Angstrom; (**c**) 2D representation of key interacting groups between GGT and probe-4.

In order to investigate the binding affinity of probe-4 with GGT, molecular docking was performed. The appreciable accuracy of the phi (φ) and psi (ψ) angles among the reaction coordinates of the targeted protein with GGT was found through a Ramachandran plot, as shown in the Figure S1 (Supporting Information). The results exhibited stable interactions of probe-4, with the active site residues of GGT having binding energy values of −8.4 kcal/mol (Figure 5a). The docking results revealed that both the hydrogen atoms of probe-4 participated in hydrogen bonding with the residues ASN401 and ASP423, while the oxygen atom of probe-4 made stable hydrogen bonds with the active residues SER452 and MET453, respectively (Figure 5b). However, some hydrophobic interactions (π-alkyl) were also shown between the phenyl ring of probe-4 and ASP422 and LEU402, respectively [31]. It is worth noting that the synthesized probe was specifically binding with one of the most important GGT active site residues. I.e., ASN401 made a hydrogen bond with one of the atoms of its substrate [32] (Figure 5b,c).

### 2.4. Bioimaging

**Figure 6:**
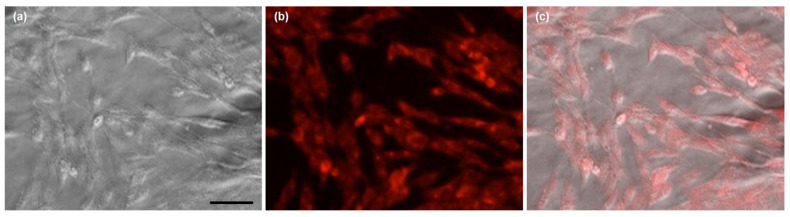
Results of bioimaging analysis upon incubation of BHK-21 cells with probe and GGT; (**a**) bright field images; (**b**) fluorescence; and (**c**) merged images; scale bar: 50 µM.

**Figure 7:**
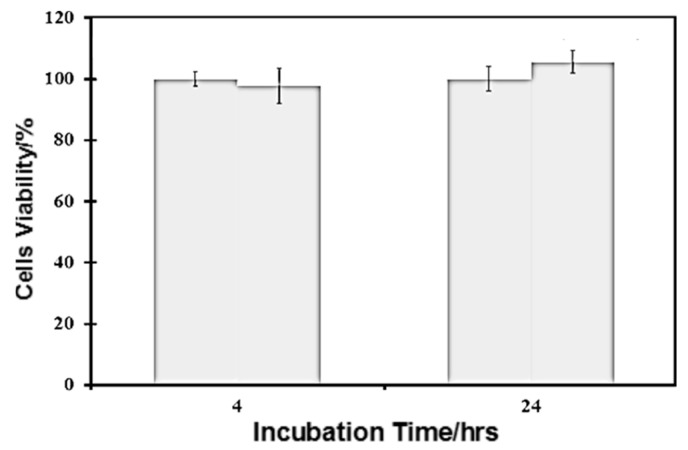
Cell viability at various incubation times following treatment with probe at concentrations of 0 and 5 µM.

The applicability of the probe to analytical tracking inside the biological media was investigated via bioimaging analysis using hamster kidney fibroblast cells (BHK-21 cells). Prior to the operation of the cells with the confocal fluorescence microscope, the incubation of the cells was performed with the 30 µM concentration of the probe at 37 °C for 3 h in the complete minimum essential media. These cells were then fed with 50 µL of enzyme from the 0.05 U/mL enzyme solutions and incubated further for 3 h, and confocal fluorescence images were recorded, which exhibited bright red fluorescence from the cells. The probe was found to have very good permeability with regards to the cellular media and throughout the entire procedure was found to be friendly with cells, without any deformation or morphological variations (Figure 6). The cellular toxicity of the probe was evaluated using the MTT assay. Briefly, the toxicity procedure was conducted via 24 h of pretreatment of the cells with the probe, and the results exhibited >97% cell viability at 5 µM concentration and ≥94% viability at 10 µM concentration. This experiment suggested the broad spectrum utility of the probe for biological systems and in-vivo monitoring (Figure 7).

## 3. Experimental

### 3.1. Substrate and Reagents

The chloride salt of rhodamine B, POCl_3_, liquid ammonia, BOC-L-glutamic acid-1-ter-butylester, HATU, DIPEA, triethylamine, KOH, NaOH, NAHCO_3_, molecular sieves, magnesium turning, and iodine pellets were purchased from Aldrich. The *γ*-glutamyltranspeptidase (GGT; EC 2.3.2.2), urease, alkaline phosphatase, acetylcholinesterase, tyrosinase and cyclooxygenase, and related enzyme assay kits were purchased from Aldrich. The solvents utilized, including ethyl alcohol, dichloromethane, acetonitrile, dimethylsulfoxide, tetrahydrofuran, ethyl acetate, *n*-hexane, chloroform and methyl alcohol, were purchased from Aldrich and Samchun.

### 3.2. Instrumentations

Aluminum pre-coated thin layer chromatographic plates (Kieselgel 60 F254) from Merck, Darmstadt, Germany were used. Melting points were recorded using a Fisher Scientific apparatus from the Hampton, NH, USA. The products were dried using a vacuum desiccator before spectroscopic analysis. A vacuum filtration assembly, including a vacuum pump and Buchner/sintered glass funnel, was utilized during filtration. Vibrational spectroscopic analysis was performed with a Shimadzu FTIR–8400S spectrometer from Japan. Bruker Avance 400 MHz NMR instruments were employed for the proton NMR analysis. Optical analysis was conducted with a Scinco absorption and emission spectrophotometer.

### 3.3. Synthesis

**Scheme 2:**
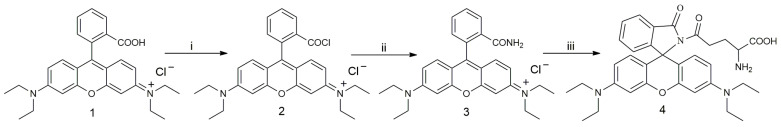
Synthesis of probe-4: (i) Phosphorous oxychloride, dichloromethane, reflux for 2 h; (ii) Liq. NH_3_, dichloromethane, stirring overnight; (iii) BOC-L-glutamic acid-1-ter-butylester, HATU, DIPEA, dichloromethane, stirring for 4 h followed by overnight stirring with TFA.

Probe-4 was synthesized starting from a chloride salt of rhodamine B, which was chlorinated by using phosphorous oxychloride as a chlorinating agent and dichloromethane as a solvent. The reactants were transformed into the chlorinated derivative **2** within 3–4 h by the equivalent treatment. The rhodamine acid chloride **2** was concentrated on reduced pressure by removing the solvent and excess chlorinating agent, and this chlorinated rhodamine B derivative was directly employed for amide synthesis upon treatment with liquid ammonia in dichloromethane. The resulting amide derivative **3** (3.32 mg in 5 mL of solvent; 1.5 milli molar; 1 eq.) was coupled with BOC-L-glutamic acid-1-ter-butylester (4.545 mg/5 mL of solvent; 3 milli molar; 2 eq.) by using HATU (5.7 g/5 mL; 3 milli molar; 2 eq.)/DIPEA (1.93 g/5 mL; 3 milli molar; 2 eq.) in dichloromethane to afford the probe-4 upon de-protection with trifluoroaceteic acid (Scheme 2) [33,34]. Supporting data related to synthesis can be found in the Supplementary Information, specifically in Figures S2–S5.

#### 3.3.1. Synthesis of 2-(3,6-Bis(diethylamino)-9H-xanthen-9-yl)benzamide (**3**)

Off-white powder; yield: 67%; R*_f_*: 0.46 (dichloromethane: methanol, 9:1); ^1^H NMR (500 M Hz, DMSO-*d_6_*) 7.83–7.82 (aromatic, 1H, m), 7.62–7.57 (aromatic, 2H, m), 6.53–6.46 (aromatic, 4 H, m), 6.44–6.37 (aromatic, 2H, m), 6.35–6.27 (aromatic, 2H, m), 3.308–3.29 (aliphatic, 8H, q, *J* = 7.5 Hz), 1.09–1.05 (aliphatic, 12H, t, *J* = 13 Hz); ^13^C NMR (125 M Hz, DMSO-*d_6_*) 188.31, 167.45, 157.72, 153.46, 153.13, 133.49, 130.73, 128.97, 128.87, 124.22, 122.90, 108.78, 97.67, 64.49, 44.25, 12.82.

#### 3.3.2. 2-Amino-5-(3′,6′-bis(diethylamino)-3-oxospiro[isoindoline-1,9′-xanthen]-2-yl)-5-oxopentanoic Acid (**4**)

Off-white amorphous solid; Yield: 64%; R_f_; 0.11 (10% dichloromethane and methanol). FT-IR (ATR, cm^−1^); 3675, 3445, 3372, 2800, 3181, 1695, 1590–1415, 1236. ^1^H NMR (400 MHz, CDCl_3_); 7.88 (2 H, doublet of doublets, *J* = 8.5 Hz), 7.42 (2H, doublet of doublets, *J* = 8.5 Hz), 7.08 (1H, multiplets), 6.41 (2H, multiplets), 6.28 (2H, multiplets), 6.21 (2H, multiplets), 5.22 (2H, singlet, amino protons), 3.25 (quartet, eight aliphatic protons of rhodamine moiety, *J* = 8.5 Hz); 3.25 (multiplet, alpha hydrogen of terminal unit); 3.25 (2H, triplet, *J* = 9 Hz), 3.08 (2H, multiplets), 1.90 (12 protons triplets, *J* = 9 Hz); ^13^C NMR (100 M Hz, CDCl_3_) *δ* 175.844, 169.490, 153.771, 153.243, 153.199, 148.846, 132.680, 130.518, 128.466, 128.092, 123.834, 122.801, 108.173, 104.941, 104.845, 97.708, 77.123, 122.801, 108.173, 104.941, 97.708, 77.123, 65.397, 50.843, 44.320, 39.989, 39.554, 21.595, 12.574; MS for C_33_H_38_N_4_O_5_ (ESI, *m*/*z*), 571 [M + H]^+^.

### 3.4. Spectroscopic and Bioimaging Analysis

A 1 mM ligand solution was prepared in ethanol for the emission and absorption measurements [35,36,37]. A total of 0.05 U/mL enzyme stock solutions were utilized for spectroscopic measurements. The sample loading was carried out by a 3 mL quartz cuvette with a 30 µM measuring concentration of the probe. The fluorescence- and absorption-related enzymatic activity was measured by using a variable concentration ranging from 10 to 100 μL of the enzyme stock solution [38,39,40]. All the spectral recordings were performed by incubating the sample at 37 degrees Celsius for 30 min prior to running with the UV and Fluorescence spectrophotometer.

### 3.5. Methodology for Docking Analysis

Autodock 4.2 Vina software (The Scripps Institute) using the Lamarckian genetic algorithm was employed to figure out the interactive behavior of the probe towards the targeted protein. The *γ*-glutamyltranspeptidase (GGT) crystal structure was retrieved from RCSB protein databank (PDB ID: 4ZBK). The protein preparation was performed by using Autodock tools, and all water molecules and in-bound ligands and heteroatoms were deleted. Then, polar hydrogens and Kollman charges were assigned, and Gasteiger charges were computed. The structure of the ligand (probe-4) was sketched on ChemDraw and was converted to the pdb format using PyMoL. The parameters utilized for the Autodock Vina run included the G.A. population size of 150 and maximum energy evolutions of 2.5 × 10^6^ generations. The grid box size was −28.91 Å, −22.97 Å, −7.59 Å along the three-dimensional coverage of the entire GGT molecule with 0.419 Å of grid point spacing. The binding energy was obtained for the best conformational pose and the interactions were analyzed with BIOVIA Discovery Studio 3.5 [41,42].

### 3.6. Bioimaging

The bioimaging analysis was performed by using hamster kidney fibroblast cells, BHK-21, which were incubated with the probe molecule in the minimum essential media for four hours at ambient temperature, and then optical measurement was carried out. The fluorescence from the cells upon reaction with GGT was recorded prior to washing with phosphate buffer saline twice, which exhibited bright red fluorescence from the cells.

## 4. Conclusions

Fluorescent materials for detecting cancer-related enzymes are vital for diagnosis and treatment. We developed a substituted rhodamine derivative to detect the enzyme *γ*-glutamyltranspeptidase (GGT). Initially, the probe is non-chromic and non-emissive due to its spirolactam form, which prevents electronic delocalization. Selective enzymatic cleavage triggers a spirolactam ring opening, allowing electronic flow across the rhodamine skeleton. This change turns the solution from colorless to intense pink, with strong UV and fluorescence signals at 551 and 598 nm. The sensor’s selectivity was tested against various human enzymes, including urease, alkaline phosphatase, acetylcholinesterase, tyrosinase, and cyclooxygenase, showing no response. Absorption and fluorescence titration analyses showed consistent spectral increases with incremental GGT addition (10–100 µL from 0.05 U/mL enzyme solutions) to the probe solution (30 µM), along with intensified pink coloration. Cellular toxicity, assessed using the MTT assay, showed >97% cell viability at 5 µM and ≥94% at 10 µM. Bioimaging of BHK-21 cells treated with the probe and GGT produced bright red fluorescence, indicating excellent cell penetration and digestion for intracellular detection. The docking results demonstrated that the hydrogen of the probe interacted with the residues ASN401 and ASP423, while the oxygen atom formed stable hydrogen bonds with the active residues SER452 and MET453. Additionally, the observation of some hydrophobic interactions was recorded among the probe’s phenyl ring and ASP422 & LEU402.

## Figures and Tables

**Table 1:** Solvent effect on optical properties of probe and its enzyme mixture.

Solvent	Optical Parameters					
	UV-Analysis			Fluorescence Analysis		
	^a^ Abs. Max.	^b^ Abs. Int.	^c^ S. Shift	^d^ Ex. Wav.	^e^ Em. Max.	^f^ Em. Int.
EtOH:H_2_O; 60%	551	0.248	47	550	598	5230
MeOH:H_2_O; 60%	551	0.248	47	550	598	5230
Ethanol	551	0.249	47	550	598	5248
Methanol	551	0.249	47	550	598	5248
DMSO	551	0.248	47	550	598	5238

^a^ Absorption maxima (nm); ^b^ Absorption itensity; ^c^ Stoke’s shift (nm); ^d^ Excitation wavelength; ^e^ Emission maxima (nm); ^f^ Emission spectral intensity (a.u).

## Data Availability

Data are contained within the article.

## Supplementary Materials

The supporting information can be downloaded at: https://www.mdpi.com/article/10.3390/molecules29194776/s1; Figure. S1: Ramachandran plot of GGT; Figure. S2. 1H NMR Spectrum of Molecule 3; Figure. S3. 13C NMR Spectrum of Molecule 3; Figure. S4. 1H NMR Spectrum of molecule 4; Figure. S5. 13C NMR Spectrum of molecule 4.
