# Supplementary material for: Turn-On Fluorescence Probe for Cancer-Related γ-Glutamyltranspeptidase Detection
Source: Molecules. 2024 Oct 9;29(19):4776. doi: 10.3390/molecules29194776 (PMC11477498; doi:10.3390/molecules29194776)
Supplement: Supplementary file 1 [file molecules-29-04776-s001.zip › molecules-3223148-supplementary.pdf]

Supporting Information

Turn-On Fluorescence Probe for Cancer Related γ-glutamyltranspeptidase Detection

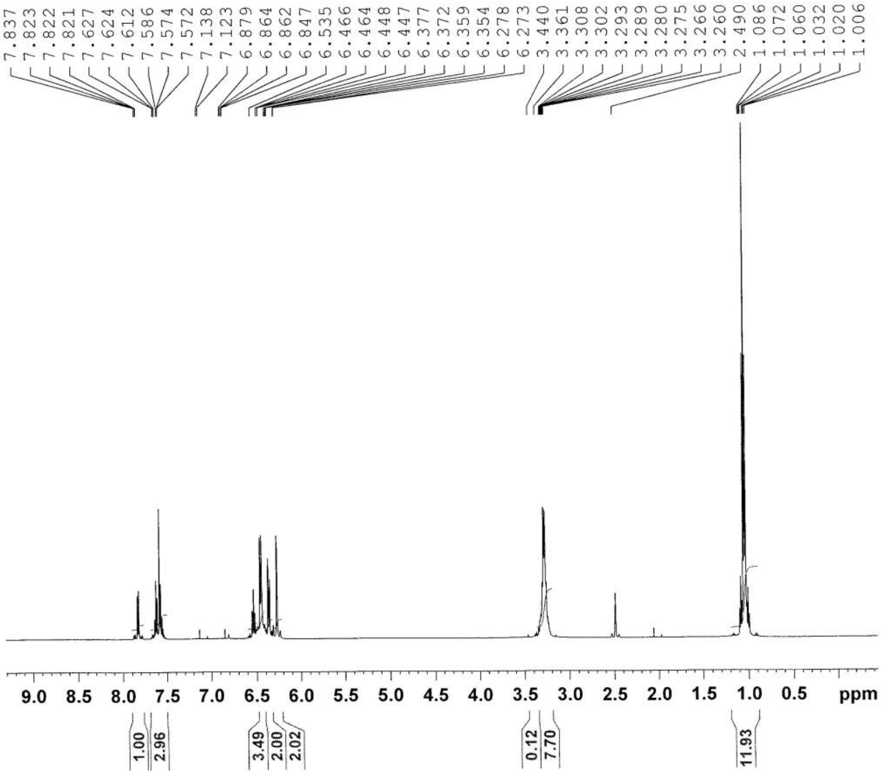

Figure S1. ^{1}H NMR Spectrum of Molecule 3

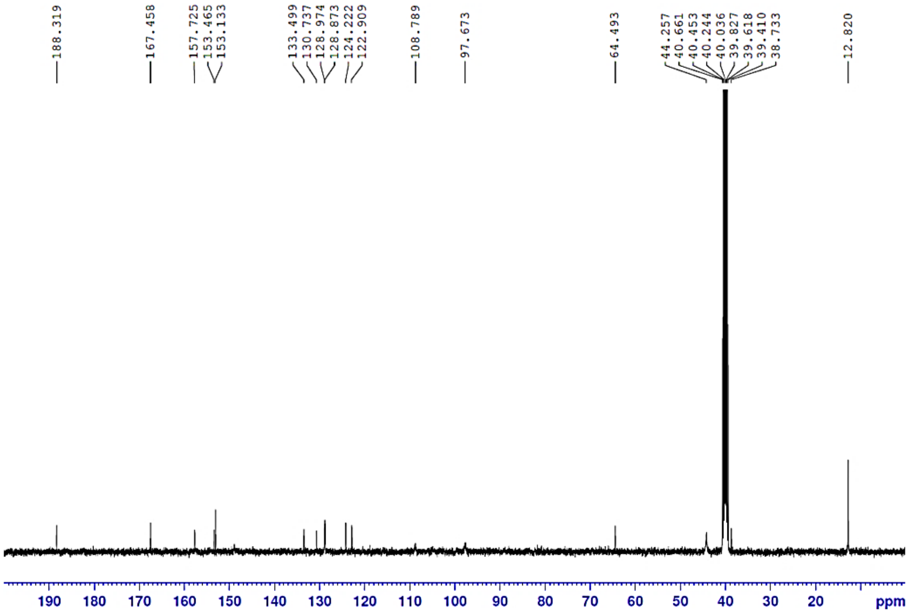

Figure S2. ^{13}C NMR Spectrum of Molecule 3

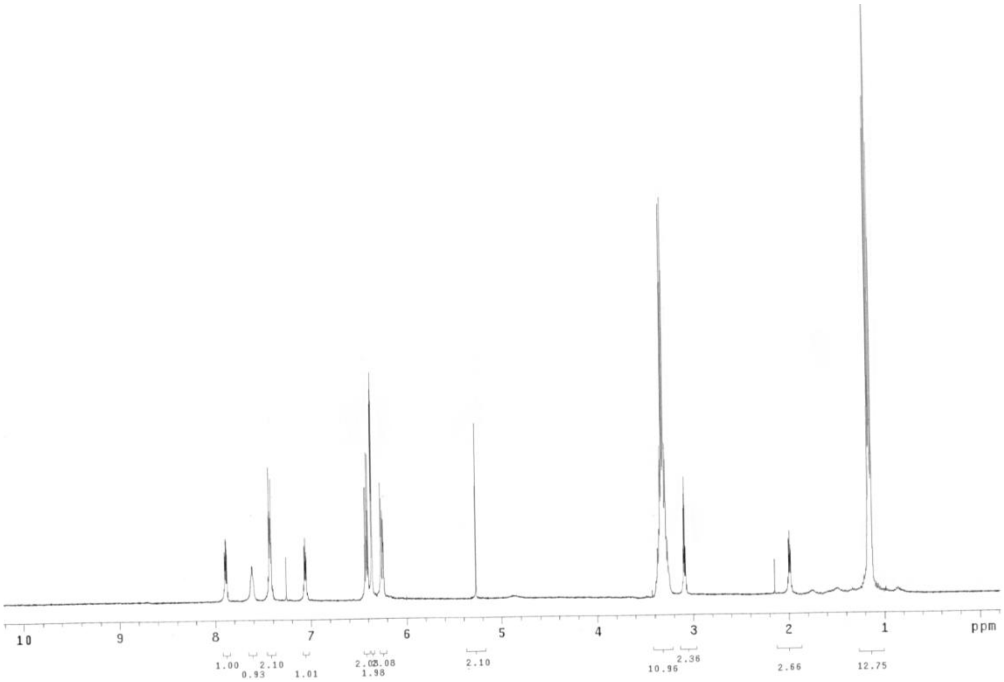

Figure S3. ^{1}H NMR Spectrum of molecule 4

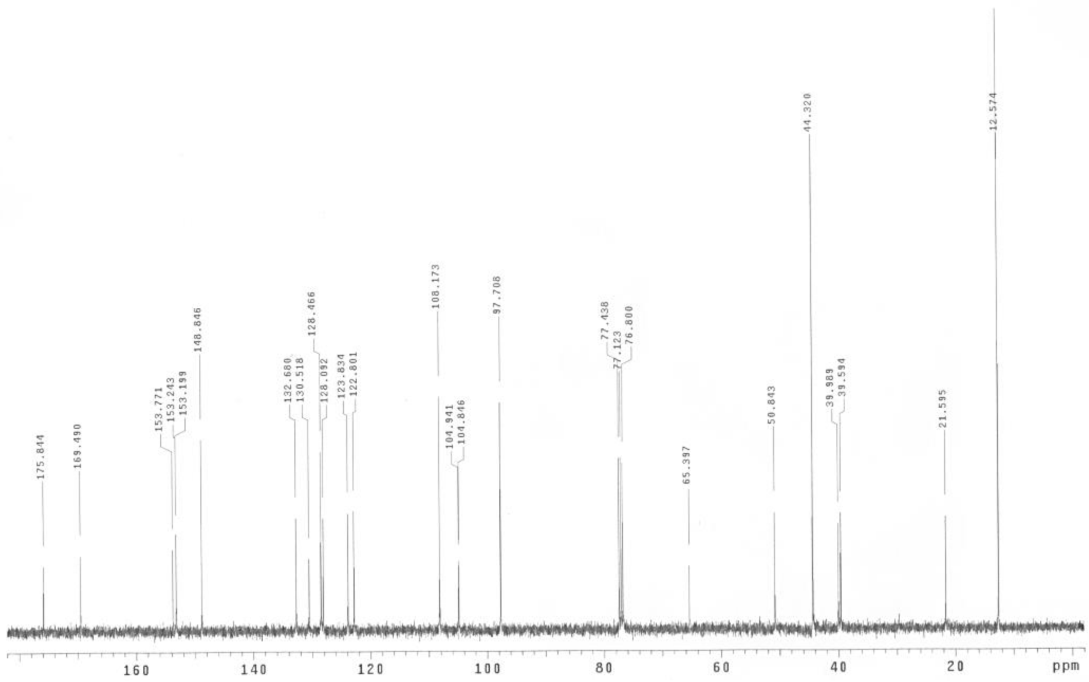

Figure S4. ^{13}C NMR Spectrum of molecule 4

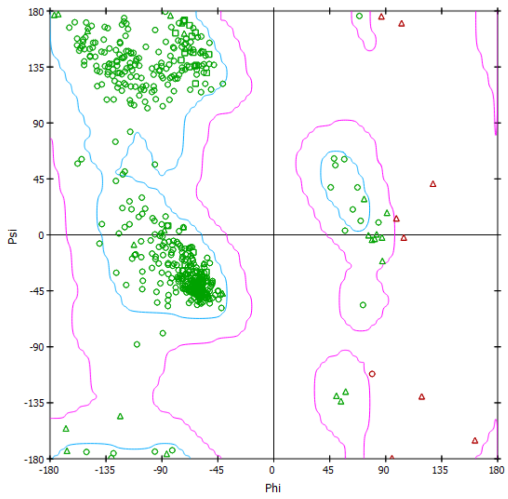

Figure S5. Ramachandran plot of GGT
